# Influence of Control Group on Effect Size in Trials of Acupuncture for Chronic Pain: A Secondary Analysis of an Individual Patient Data Meta-Analysis

**DOI:** 10.1371/journal.pone.0093739

**Published:** 2014-04-04

**Authors:** Hugh MacPherson, Emily Vertosick, George Lewith, Klaus Linde, Karen J. Sherman, Claudia M. Witt, Andrew J. Vickers, on behalf of the Acupuncture Trialists' Collaboration

**Affiliations:** 1 Department of Health Sciences, University of York, York, United Kingdom; 2 Memorial Sloan-Kettering Cancer Center, New York, New York, United States of America; 3 Faculty of Medicine, Primary Care and Population Sciences, University of Southampton, Southampton, United Kingdom; 4 Institute of General Practice, Technische Universität München, Munich, Germany; 5 Group Health Research Institute, Seattle, Washington, United States of America; 6 Center for Complementary and Integrative Medicine, University Hospital Zurich, Zurich, Switzerland; 7 Institute for Social Medicine, Epidemiology and Health Economics, Charité - Universitätsmedizin, Berlin, Germany; National Taiwan University, Taiwan

## Abstract

**Background:**

In a recent individual patient data meta-analysis, acupuncture was found to be superior to both sham and non-sham controls in patients with chronic pain. In this paper we identify variations in types of sham and non-sham controls used and analyze their impact on the effect size of acupuncture.

**Methods:**

Based on literature searches of acupuncture trials involving patients with headache and migraine, osteoarthritis, and back, neck and shoulder pain, 29 trials met inclusion criteria, 20 involving sham controls (n = 5,230) and 18 non-sham controls (n = 14,597). For sham controls, we analysed non-needle sham, penetrating sham needles and non-penetrating sham needles. For non-sham controls, we analysed non-specified routine care and protocol-guided care. Using meta-regression we explored impact of choice of control on effect of acupuncture.

**Findings:**

Acupuncture was significantly superior to all categories of control group. For trials that used penetrating needles for sham control, acupuncture had smaller effect sizes than for trials with non-penetrating sham or sham control without needles. The difference in effect size was −0.45 (95% C.I. −0.78, −0.12; p = 0.007), or −0.19 (95% C.I. −0.39, 0.01; p = 0.058) after exclusion of outlying studies showing very large effects of acupuncture. In trials with non-sham controls, larger effect sizes associated with acupuncture vs. non-specified routine care than vs. protocol-guided care. Although the difference in effect size was large (0.26), it was not significant with a wide confidence interval (95% C.I. −0.05, 0.57, p = 0.1).

**Conclusion:**

Acupuncture is significantly superior to control irrespective of the subtype of control. While the choice of control should be driven by the study question, our findings can help inform study design in acupuncture, particularly with respect to sample size. Penetrating needles appear to have important physiologic activity. We recommend that this type of sham be avoided.

## Introduction

One of the challenges of conducting a non-pharmacological clinical trial is choosing an appropriate control intervention. The simplest control arm is to offer patients routine clinical care without the experimental treatment. This controls for the expected course of the disease. However, the control arm can also be designed to control for other factors, for example, the non-specific effects associated with the time and attention that a patient receives from a clinician.

The choice of control is particularly problematic in acupuncture, which has seen a large increase in published trials in recent years [1]. In an individual patient data meta-analysis of high quality trials conducted by the Acupuncture Trialists' Collaboration [2], acupuncture reduced pain scores by 0.15 to 0.23 standard deviations in comparison to sham (placebo) acupuncture. When the control group did not involve sham, effect sizes ranged from 0.42 to 0.57 [2]. Yet within the general categories of control - those with sham and those without sham - there were marked differences in the exact nature of the intervention received in the control group. For example, trials with sham control included those with acupuncture needles inserted at points not thought to be active, needles that did not penetrate the skin, and non-needle approaches, such as detuned electrical devices. For trials without sham control, the control group in some were simply advised to “avoid acupuncture”; in other trials, both acupuncture and control groups were offered additional treatment, such as physical therapy for back pain.

In this paper, we aim to conduct an analysis of the Acupuncture Trialists' Collaboration dataset to determine how trial results vary by type of control. Specifically, we sought to determine the extent that effect sizes varied depending on whether needles were used for sham acupuncture, whether they penetrated the skin, and whether they were placed at or away from true acupuncture points. We also sought to determine whether there was variation in the effects of acupuncture associated with controls that did not involve sham, comparing “routine care”, such as rescue medication made available to patients in both arms of the trial, with “protocolled care” where the control treatment was a standard care specified in the study protocol. Establishing effect sizes associated with commonly used types of controls will be of value in informing future clinical trial design for acupuncture, as well as helping the interpretation of published trial results.

## Methods

### Included Trials

Trials included in these analyses were identified through a systematic literature review that has been previously described [2]. The initial search was to November 2008, followed by a subsequent one conducted in December 2010. The searches included trials of acupuncture in four specified chronic pain conditions – non-specific musculoskeletal pain, shoulder pain, osteoarthritis, and chronic headache – where allocation concealment was determined unambiguously to be adequate. For trials of musculoskeletal pain, it was additionally specified that the current episode of pain must be of at least four weeks' duration. The search resulted in the identification of 31 trials.

### Data Acquisition

Individual patient data were obtained from 29 trials. Data on the trial-level characteristics of the controls were obtained directly from trialists. Twenty trials with 5,230 patients had controls in the form of a sham acupuncture arm (Table 1), and 18 trials with 14,597 patients had non-sham controls (Table 2).

**Table 1:** Sham Acupuncture-Controlled Trials, by Types of Sham Control.

Needle Used?	Penetrating?	True Acupuncture Points?	Depth of Insertion?	Trials
Yes	Yes	No	Superficial	Linde (2005) [3], Melchart (2005) [25], Diener (2006) [6], Scharf (2006) [26], Haake (2007) [11], Endres (2007) [27], Witt (2005) [28], Brinkhaus (2006) [29]
Yes	Yes	No	Deep	Berman (2004) [5] *
Yes	No	No	N/A	Vas (2008) [14]
Yes	No	Yes	N/A	Foster (2007) [24], Guerra (2004) [30], Kennedy (2008) [31], Kleinhenz (1999) [4], Vas (2004) [12], Vas (2006) [13]
No	No	No	N/A	Carlsson (2001) [9], Kerr (2003) [8]
No	No	Yes	N/A	Irnich (2001) [7], White (2004) [32]

*In this trial, penetrating needles were used on non-acupuncture points and non-penetrating needles were used on true acupuncture points. For the main analysis, we considered this trial as penetrating-needle sham used on non-acupuncture points.

**Table 2:** Types of non-sham control group by trial: categorised as “protocolled care” or “routine care”.

Trial	Control Group	Type of Control Group
Foster (2007) [24]	Advice and exercise: All three arms of the trial received advice and exercise. Patients received leaflet with information on knee osteoarthritis. Patients on NSAID therapy were allowed to continue with stable dose. Individualized exercises of progressive intensity for lower limb stretching, strengthening and balance (up to six 30-minute sessions over six weeks). Patients in the control arm did not receive verum or sham acupuncture.	Protocolled
Linde (2005) [3], Melchart (2005) [25]	Waiting list control: Control patients were not permitted to have prophylactic treatment for 12 weeks. All patients were allowed to treat acute headache as necessary (following current guidelines).	Routine
Thomas (2006) [33], Salter (2006) [34], Vickers (2004) [23]	General practitioner care: All patients received NHS treatment according to general practitioner's assessment and recommendation. Control patients did not receive acupuncture or any other specified interventions.	Routine
Berman (2004) [5]	Education-attention control: Patients in this arm attended six two-hour group sessions based for arthritis self-management, and received periodic educational materials by mail. Patients in the acupuncture and sham acupuncture arms did not participate in this intervention.	Routine
Cherkin (2001) [35]	Self-care education: Patients in this group received a book with information about back pain, treatment, improving quality of life and coping with emotional and interpersonal issues surrounding back pain. Patients also received two professionally-produced videos which addressed self-management of back pain and demonstrated exercises. Patients in the acupuncture and massage groups did not receive this educational material.	Routine
Scharf (2006) [26]	Conservative therapy: Patients in the conservative therapy group had 10 visits with physicians and received prescriptions for either diclofenac (up to 150 mg/day) or rofecoxib (25 mg/day) up to week 23. Patients in this group who had “partially successful” results were offered the choice of attending an additional five visits. Patients in the verum acupuncture and sham acupuncture groups were permitted to take up to 150 mg/day of diclofenac for the first two weeks and a total of 1 g of diclofenac during the rest of the study. Patients in both acupuncture groups and in the conservative management group received up to six sessions of physiotherapy. All patients were prohibited from taking any analgesics other than diclofenac and rofecoxib and any corticosteroids.	Protocolled
Diener (2006) [6]	Standard migraine treatment: Control group patients were treated according to the guidelines of the German Migraine and Headache Society. Patients had six to seven visits in which standard treatment was established. First choice of treatment was beta blockers, followed by flunarizine, and then valproic acid. Acute medication use was permitted in all groups.	Protocolled
Haake (2007) [11]	Conventional therapy: Patients in the conventional therapy group were treated according to German guidelines. Conventional therapy patients had 10 visits with physician or physiotherapist where physiotherapy, exercise and/or similar treatments were offered. Patients in all three arms were permitted to take NSAIDs up to the maximum daily dose.	Protocolled
Williamson (2007) [36]	Education and exercise: Patients in the control group were told they were in the “home exercise” group and received an exercise and advice leaflet.	Routine
Witt (2005) [28], Brinkhaus (2006) [29]	Waiting list control: Patients in the waiting list control group received no acupuncture treatment for eight weeks after randomization. All patients were allowed oral NSAIDs for pain as rescue medication. All patients were prohibited from taking corticosteroids or pain medication that acted on the central nervous system.	Routine
Witt (2006 – OA) [37], Witt (2006 – LBP) [38]	Conventional treatment: Patients in the control group were not allowed to use any kind of acupuncture during the first three months. All patients were allowed to use additional conventional treatments as needed.	Routine
Jena (2008) [39], Witt (2006 – Neck Pain) [40]	Conventional treatment: Patients in the control group were not allowed to use any kind of acupuncture during the first three months. All patients were allowed to use additional conventional treatments as needed.	Routine

### Outcome

The primary outcome used for this analysis was the primary pain endpoint as defined by the study authors. Where multiple criteria were considered in the primary outcome or if the primary outcome was inherently categorical, we used a continuous measure of pain intensity measured at the same time point as the original primary outcome. To make the various outcome measurements comparable between different trials, the primary endpoint of each was standardized by dividing by pooled standard deviation.

### Types of Sham Acupuncture Controls

The characteristics we aimed to study in those trials with a sham acupuncture control group included whether or not a needle was used, whether a needle that penetrated the skin was used, whether sham was performed on true acupuncture points or non-acupuncture points, and whether needle insertion was deep or superficial. Information on acupuncture characteristics was obtained from the trial manuscript supplemented by a questionnaire sent to trialists.

Trials were classified as “needle sham” if it was reported that either a penetrating or non-penetrating needle was used for sham acupuncture. A non-penetrating needle is a device specially developed for acupuncture research in which the needle retracts into the handle rather than penetrating the skin; however, the pressure of the needle against the skin is a very similar sensation to insertion. “Non-needle sham” included trials using non-needle methods of sham acupuncture, such as an inactivated laser or transcutaneous electric nerve stimulation (TENS) device. Needle sham trials were further classified as to whether or not the needle used in the sham acupuncture group penetrated the skin. Penetrating needles were almost always inserted at locations away from true acupuncture points (thereby investigating point location) while non-penetrating sham needles were either applied at the same points as in the true acupuncture group (testing exclusively skin penetration and not location) or at non-acupuncture points (investigating penetration and location simultaneously). For example, in the trial of Linde et al [3], needles were inserted superficially away from true acupuncture points; in contrast, the sham technique in the Kleinhenz et al [4] trial consisted of a special needle that retracted into the handle rather than penetrating through the skin at true acupuncture points.

We initially planned to investigate two other features of sham control: whether the depth of insertion for penetration was categorized by trialists as superficial or deep and whether sham was applied at or away from true acupuncture points. However, as shown in Table 1, only one trial reported using deep insertion in sham acupuncture [5]. For point location, there was strong collinearity with sham technique, with only techniques avoiding skin penetration using true acupuncture points.

As a sensitivity analysis, we re-analyzed the data excluding four trials which were determined by consensus among external reviewers as having an “intermediate likelihood of unblinding” [6] [7] [8] [9]. However, after excluding these trials, only one remaining trial used non-needle sham acupuncture, limiting our ability to use meta-regression.

### Types of Non-sham Controls

Trials that included controls without sham were categorized into two types: “routine care” and “protocolled care.” Trials were identified as “routine care” if patients in both treatment and control groups had access to non-specified care as needed, such as rescue medications or other conventional care, but the use of such treatment was at the discretion of patients and doctors, with no specification in the protocol as to what treatments patients could receive. If protocols proscribed some treatments, such as surgery, but did not make specific recommendations as to allowable treatments, trials were defined as “routine care”. Control groups where treatment consisted of information or education given to a patient (“attention control”) were also considered to be routine care control groups. Trials were considered to be “protocolled care” if the care in the control group was specified in the study protocol. This was typically when the acupuncture group and the usual care control group both received an additional non-acupuncture treatment that was specifically indicated as part of the trial protocol. For example, trials that studied the effect of acupuncture and physical therapy compared to physical therapy alone were categorized as protocolled care.

In two trials there was a disagreement about whether the close specification of medication and other treatment in the control group constituted “protocolled care” or an active control group, which are excluded from analyses as per the review protocol [10]. The trial of acupuncture for migraine by Diener et al. [6] had a control group in which patients received standard pharmacological therapy for migraine prophylaxis. However, acupuncture and sham acupuncture groups did not receive prophylactic medication. The trial of acupuncture for lower back pain by Haake et al. [11] offered “routine management” up to and including physiotherapy, drugs and exercise to control group patients. Acupuncture and sham acupuncture patients had the same access to rescue medication as the “routine management” group, but did not have access to the same physiotherapy sessions or exercise consultations with physicians.

### Statistical Methods

We used random-effects meta-regression to test the effect of each characteristic of sham acupuncture on the main effect estimate using the Stata command *metareg*. This command was also used to run a random-effects meta-regression to test the effect of routine versus protocolled care on the main effect estimate for usual care control groups. The main effect estimate of each trial was determined using linear regression, and the coefficient and standard error for each trial were entered as the dependent variable in the random-effects meta-regression.

A sensitivity analysis was performed excluding three trials by Vas et al. [12] [13] [14]. In our initial publication on effect size [2], we reported that these trials have very much larger effect sizes than average and that their exclusion resulted in heterogeneity becoming non-significant in the comparisons between acupuncture and sham. The trial of acupuncture for knee osteoarthritis by Berman et al. [5] used a combined insertion and non-insertion method for sham acupuncture. As a sensitivity analysis, we performed the analysis with this trial reclassified as using non-penetrating needles on true acupuncture points as well. We also excluded trials where the risk of bias from unblinding was not classed as being low. As a final sensitivity analysis of non-sham controlled trials, we excluded the trials by Haake et al. [11] and Diener et al. [6] for which there was disagreement as whether the control arm constituted active control, which is not eligible for analysis [10]. All analyses were conducted using Stata 12 (Stata Corp., College Station, TX).

## Results

### Sham acupuncture controls

Trial-level characteristics for sham-controlled trials are described in Table 3. The majority of sham-controlled trials (80%) used needle-based sham acupuncture. The number of trials using penetrating or non-penetrating needles was similar: seven trials used non-penetrating needles and nine trials used penetrating needles. All trials using penetrating needles placed these outside true acupuncture points, while only one of seven trials using non-penetrating needles did so.

**Table 3:** Trial-level Characteristics for Trials with Sham Acupuncture Control Groups, N = 20.

Needle Used	
Yes	16 (80%)
No	4 (20%)
Penetrating Needle Used	
Yes	9 (45%)
No	7 (35%)
Non-needle	4 (20%)
True Acupuncture Points Used	
Yes	8 (40%)
No	12 (60%)
Superficial or Deep Sham	
Superficial	8 (40%)
Deep	1 (5%)
Non-penetrating sham	11 (55%)
Pain Type	
Low Back Pain	5 (25%)
Migraine	2 (10%)
Neck	3 (15%)
Osteoarthritis	5 (25%)
Shoulder	3 (15%)
Tension-type Headache	2 (10%)

Frequency (%).

Table 4 shows the effect sizes of sham-controlled acupuncture trials categorized by the type of sham. Acupuncture is significantly superior to sham irrespective of the type of sham control, both in the main analysis and in a sensitivity analysis excluding outlying studies. Table 4 also includes the results of the primary sensitivity analyses that excluded the Vas trials, which we had previously found to be outliers [2].For example, not only was the effect size of the Vas trial for neck pain [13] about five times greater than the meta-analytic estimate, but between-trial heterogeneity was no longer statistically significant after excluding the Vas trials. Using the same rationale for exclusions, overall we found larger effect sizes were associated with acupuncture vs. non-penetrating sham needles (0.43; 95%CI: 0.01, 0.85) than vs. penetrating sham needles (0.17; 95%CI: 0.11, 0.23) although the difference between groups did not reach conventional levels of statistical significance.

**Table 4:** Effect size of acupuncture compared to type of sham acupuncture control.

	Main Analysis		Excluding Vas et al. trials [12] [13] [14]	
	Number of Trials	Effect Size	Number of Trials	Effect Size
Needle sham	16	0.42 (0.19, 0.66)	13	0.22 (0.11, 0.33)
Non-needle sham	4	0.38 (0.19, 0.57)	4	0.38 (0.19, 0.57)
Non-penetrating needle	7	0.76 (0.31, 1.21)	4	0.43 (0.01, 0.85)
Penetrating needle	9	0.17 (0.11, 0.23)	9	0.17 (0.11, 0.23)
Non-needle and non-penetrating needle	11	0.63 (0.33, 0.94)	8	0.40 (0.18, 0.62)

Estimates obtained using meta-regression.

Statistical comparisons between types of sham are given in Table 5, which shows the results of the random-effects meta-regression for sham-controlled trials. While trials that used needles as sham did not differ significantly from trials with non-needle sham (p≥0.2 for all comparisons), there is clear evidence of a greater effect size when acupuncture is compared against non-penetrating sham than when compared to penetrating sham. Trials using a penetrating needle had an effect size of −0.21 (95% C.I. −0.41, −0.01) standard deviations lower than trials that did not use a needle sham (p = 0.036). Trials that used penetrating needles for sham control had smaller effect sizes than those with non-penetrating sham or sham control without needles. The difference in effect size was −0.45 (95% C.I. −0.78, −0.12; p = 0.007). For the sensitivity analysis that excluded the Vas trials, this effect size reduced to −0.19 (95% C.I. −0.39, 0.01; p = 0.058). There were no significant differences between non-penetrating needles and sham techniques that did not involve needling.

**Table 5:** Difference in effect sizes between types of sham control. Estimates obtained using meta-regression.

	Main Analysis			Excluding Vas et al. trials [12] [13] [14]		
	No. of Trials*	Change in Effect Size	p value	No. of Trials*	Change in Effect Size	p value
Needle vs. Non-needle sham	16 vs. 4	0.02 (−0.49, 0.53)	0.9	13 vs. 4	−0.17 (−0.43, 0.09)	0.2
Non-penetrating needle vs. Non-needle sham	7 vs. 4	0.35 (−0.28, 0.99)	0.3	4 vs. 4	0.01 (−0.45, 0.47)	1
Penetrating needle vs. Non-penetrating needle	9 vs. 7	−0.57 (−0.96, −0.18)	0.004	9 vs. 4	−0.19 (−0.47, 0.08)	0.2
Penetrating needle vs. Non-needle sham	9 vs. 4	−0.21 (−0.41, −0.01)	0.036	9 vs. 4	−0.21 (−0.41, −0.01)	0.036
Penetrating needle vs. Non-needle or Non-penetrating needle	9 vs. 11	−0.45 (−0.78, −0.12)	0.007	9 vs. 8	−0.19 (−0.39, 0.01)	0.058

*The number listed in the top row is the number of trials in the first comparison group. The number of trials listed in the bottom row is the number of trials in the second comparison group. For example, there were 16 needle sham-controlled trials and 4 non-needle sham-controlled in the main analysis.

In further sensitivity analyses, reclassification of the Berman trial had little effect on our results. For example, the comparison of penetrating needle vs. non-needle or non-penetrating needle gave a difference in effect size of 0.18 rather than 0.19 and a p value of 0.057 rather than 0.058. Excluding the four trials that were classified as “intermediate risk of unblinding” did not significantly change the effect sizes or p-values for most analyses. For example, the difference in effect size and 95% confidence interval when comparing penetrating needle sham to non-penetrating needle sham was −0.57 (−0.96, −0.18) in the main analysis, while the difference in effect size and 95% confidence for the same comparison was −0.57 (−0.98, −0.15) after excluding these four trials. However, the comparison with non-needle-based sham acupuncture included only one trial using non-needle sham. As a result, the standard error becomes much larger, and the p value non-significant. Excluding the trials at intermediate risk of unblinding and the outlying Vas trials gave very similar results to the analyses excluding the outlying Vas trials alone. For example, the central estimate for the comparison of penetrating needle with non-needle sham changes from −0.21 to −0.18. Statistical significance was lost, presumably because of the limited number of trials remaining in the analysis (see Table S1, Supporting Information).

### Non-sham controls

Trial-level characteristics for trials without sham controls are described in Table 2. The majority of these trials (72%) were classified as routine care. Table 6 provides further details of the control groups, separately by pain type.

**Table 6:** Trial-level Characteristics for Trials with Non-sham Control Groups, N = 18.

Pain Type	Routine Care	Protocolled Care	Total
Headache	2	0	2
Migraine	1	1	2
Tension-Type Headache	1	0	1
Osteoarthritis	4	2	6
Lower Back Pain	3	2	5
Neck Pain	2	0	2
Total	13 (72%)	5 (28%)	18 (100%)

Frequency (%).

The effect size for acupuncture in trials with routine care control (0.55, 95% CI 0.40, 0.70) was larger than when acupuncture was compared against protocolled care (0.29, 95% CI 0.01, 0.58). Although the difference in effect size was large, it was not significant (difference in effect size  = 0.26, 95% CI −0.05, 0.57, p = 0.1). Removing the two studies [6] [11] in the sensitivity analysis had little effect on the effect size estimate (0.25, 95% CI −0.26, 0.76) for the comparison with protocolled care. The difference in effect size between trials utilizing protocolled vs. routine care was also similar (0.29, 95% CI −0.13, 0.72).

## Discussion

### Principal findings

Acupuncture was significantly superior to sham irrespective of the type of sham control and superior to non-sham control irrespective of whether that constituted routine or protocolled care. That said, there were differences in effect sizes between trials with different control conditions. With regard to the types of sham control, we found that sham controls involving penetrating needles had smaller effect sizes than trials that did not use a needle control or where the needles in the control group did not penetrate the skin. An important implication is that the central estimates from our meta-analysis [2] may have underestimated the effects of acupuncture compared to sham. With regard to non-acupuncture controls, we found evidence that the effect size of acupuncture when compared to protocolled care is smaller than when compared to the less intensive routine care, although differences did not reach statistical significance.

There are two possible explanations for the differences in effect size by type of sham control: bias from unblinding and physiologic activity. It is plausible that penetrating needles are more credible to patients than non-penetrating approaches, such that patients are less likely to give biased responses on pain questionnaires. That said, there is no evidence in favor of such a hypothesis and considerable evidence against. In particular, the most common form of non-penetrating needle used was the “Streitberger” needle that has been carefully validated as a credible placebo in an empirical study. Indeed, study participants were unable to distinguish between the Streitberger needle and true acupuncture even when subject to both in crossover fashion [15]. The other explanation for our findings is that penetrating needles have important physiologic activity, that is, inserting an acupuncture needle superficially away from an acupuncture point may be less effective than deep insertion at a correct location, but nonetheless has some therapeutic activity against pain [16] [17].

### Relationship to the literature

There has been considerable interest in the literature regarding the appropriate choice of placebo controls for non-pharmacological therapies. One approach has been to investigate trials that included a placebo arm and a no-treatment arm, and then compare outcomes between these two, and in this way explore variations in the impact of the different types of placebo. An example of this is a Cochrane review of placebo controls covering a wide range of trials for different conditions, including some acupuncture trials [18]. In a sub-group analysis the authors found that trials using “physical placebos” (including sham acupuncture) were associated with greater placebo effects than trials with pharmacological placebos [18]. This finding is consistent with the results of a trial that was specifically designed to compare a sham device (sham acupuncture) with an inert pill, the sham device being associated with a greater reduction of self-reported pain [19]. These results provide supportive evidence for our finding that different types of sham control lead to different estimates of treatment effects.

The data from the above Cochrane review of placebo controls were re-analysed by a different group of authors who observed that sham acupuncture interventions vs. no treatment have larger effects than other “physical placebos” vs. no treatment [20]. In a sub-analysis that is similar to what we report in this paper, they found that the standardised effect for acupuncture versus sham was similar for trials using penetrating sham needling (−0.43; 95%CI: −0.59, 0.28) compared to trials using non-penetrating sham (−0.37; 95%CI: −0.70, 0.04) [20]. By contrast, we found significantly smaller effect sizes when acupuncture was compared to sham acupuncture with penetrating needles (0.17; 95%CI: 0.11, 0.23) than when compared to non-penetrating needles (0.43; 95%CI: 0.01, 0.85). The differences might be explained by differences in the trials included - our data involved only chronic pain trials of methodologically high quality – and the greater precision afforded by individual patient data meta-analysis: note that the wide confidence intervals in the Cochrane data are consistent with the main estimates from the current analysis.

### Study strengths and limitations

Combining patient data from 29 high-quality trials in a single database provides us for the first time with sufficient power to explore the role of controls in trials of acupuncture for chronic pain, because the power of meta-regression is strongly influenced by the number of trials and their variation. We were unable to address questions as to the depth and location of sham needle placement as only one trial used deep sham needle insertion and all sham-controlled trials that used true acupuncture points avoided penetrating needles. While the difference in effect size between routine and protocolled care is large and in the direction expected, it is associated with wide confidence intervals. Partially, this is due to the wide variety of non-sham controls, and the difficulty we had in categorizing them.

Even with this large dataset we do not have a full understanding of the different physiologic and psychologic effects of sham acupuncture. One limitation within the field generally is that the mechanisms for a persistent effect of acupuncture on chronic pain are incompletely understood and therefore we have no clear idea of whether a sham control inadvertently activates these mechanisms or not. This lack of understanding about the physiological mechanisms of acupuncture limits any firm conclusions we can draw regarding the extent that any of the sham controls discussed above can be considered as a true ‘placebo’. Moreover when implementing sham acupuncture trials, the outcome may also be influenced by factors not included in our analysis, such as the believability of the control, prior knowledge of patients about acupuncture, whether the true acupuncture group was treated identically, the extent that practitioners were able to maintain equipoise, and practical implementation issues, such as how carefully the ring that comes with the Streitberger needle [15] was taped in place.

### Implications for research

The research question remains the primary determinant on choice of control. In a strategy document developed with a range of collaborators using consensus methods, a useful distinction has been drawn between efficacy trials that seek to determine whether there are specific effects beyond the placebo in an ideal treatment environment, and effectiveness trials that seek to determine the overall impact of acupuncture in which specific and non-specific effects are combined [21]. Moreover research questions investigating the value of specific point location need to have sham needles located away from true acupuncture points while research questions testing skin penetration require non-penetrating sham needle controls applied at the same points as in the true acupuncture group.

The choice of a sham acupuncture control needs to be informed by consideration of the likely impact of the sham intervention. In the past, judgments on this have often used expert opinion on putative physiological activity of a sham control, even though we have yet to understand the mechanism(s) of the action of acupuncture [17]. A number of commentators have speculated that penetrating sham needling may be physiologically active and thus be an inappropriate sham control [16]. Our results provide support for this contention, suggesting that needle penetration should be avoided as a sham technique to control for non-specific effects associated with acupuncture in trials involving chronic pain patients. However sham acupuncture involving penetrating needles may well have a place when addressing questions of point specificity in explanatory trials. We are more cautious with regard to recommending the use of non-penetrating needles. Many forms of Japanese acupuncture use shallow insertion or non-insertion (the *toya hari* method) [22]. Using non-penetrating needles in controlled trials is not without its challenges: although apparently less active than other types of sham, we cannot assume that non-penetrating needles have complete physiologic inactivity; furthermore, there are practical questions regarding whether to enroll only acupuncture-naïve patients and whether practitioners can maintain equipoise in large trials over reasonable periods of time.

When sham acupuncture is not used, the choice of control is clearly driven by the research question. For instance, in the UK National Health Service (NHS) trial of acupuncture for chronic headache, the study question of Vickers et al was related to the effects of making acupuncture more widely available in primary care, a pragmatic comparison of “use acupuncture” and “avoid acupuncture” [23]. On the other hand, Foster et al. were interested in the impact of acupuncture when added to an existing rehabilitation program [24]. Yet our findings have clear implications for sample size calculations, with larger sample sizes needed in trials where care in the control arm is carefully specified.

## Conclusion

From a large database of individual patient data from high-quality randomized trials, we found acupuncture to be significantly superior to control irrespective of the subtype of control. When compared against sham, trials with penetrating needles reported lower effect sizes for acupuncture than trials with non-penetrating needles or those that used non-needle sham. This suggests that penetrating needles have important physiologic activity, even when inserted superficially away from true acupuncture points. Accordingly, we recommend that this type of sham be avoided. In trials without sham control, we found that the effect size likely depends on the intensity of treatment in the control group, with smaller differences between acupuncture and protocol guided programs of treatment than between acupuncture and routine care. While the choice of control should be driven by the study question, these findings can help inform study design in acupuncture, particularly with respect to sample size.

## Supporting Information

Table S1 **Sensitivity analyses.**
(DOCX)

## References

[1] Han JS, Ho YS (2011) Global trends and performances of acupuncture research. Neurosci.Biobehav.Rev 35(1873–7528): 680–7. 20800613 10.1016/j.neubiorev.2010.08.006

[2] Vickers AJ, Cronin AM, Maschino AC, Lewith G, MacPherson H, et al (2012) Acupuncture for Chronic Pain: Individual Patient Data Meta-analysis.x. Neurosci.Biobehav.Rev 172(19): 1444–53. 10.1001/archinternmed.2012.3654 PMC3658605 22965186

[3] Linde K, Streng A, Jurgens S, Hoppe A, Brinkhaus B, et al (2005) Acupuncture for patients with migraine: a randomized controlled trial. JAMA 293: 2118–25. 15870415 10.1001/jama.293.17.2118

[4] Kleinhenz J, Streitberger K, Windeler J, Gussbacher A, Mavridis G, et al (1999) Randomised clinical trial comparing the effects of acupuncture and a newly designed placebo needle in rotator cuff tendinitis. Pain 83: 235–41. 10534595 10.1016/s0304-3959(99)00107-4

[5] BM, Lao L, Langenberg P, Lee WL, Gilpin AMK, et al (2004) Effectiveness of acupuncture as adjunctive therapy in osteoarthritis of the knee: a randomized, controlled trial. Ann Intern Med 141: 901–10. 15611487 10.7326/0003-4819-141-12-200412210-00006

[6] Diener HC, Kronfeld K, Boewing G, Lungenhausen M, Maier C, et al (2006) Efficacy of acupuncture for the prophylaxis of migraine: a multicentre randomised controlled clinical trial. Lancet Neurol 5: 310–6. 16545747 10.1016/S1474-4422(06)70382-9

[7] D, Behrens N, Molzen H, Konig A, Gleditsch J, et al (2001) Randomised trial of acupuncture compared with conventional massage and “sham” laser acupuncture for treatment of chronic neck pain. BMJ 322(322(7302)): 1574–8. 11431299 10.1136/bmj.322.7302.1574 PMC33515

[8] DP, Walsh DM, Baxter D (2003) Acupuncture in the management of chronic low back pain: a blinded randomized controlled trial. Clin J Pain 19(6): 364–70. 14600536 10.1097/00002508-200311000-00004

[9] Carlsson CP, Sjölund BH (2001) Acupuncture for chronic low back pain: a randomized placebo-controlled study with long-term follow-up. Clin J Pain 17(4): 296–305. 11783809 10.1097/00002508-200112000-00003

[10] Vickers AJ, Cronin AM, Maschino AC, Lewith G, MacPherson H, et al (2010) Individual patient data meta-analysis of acupuncture for chronic pain: protocol of the Acupuncture Trialists' Collaboration. Trials 11: 90. 20920180 10.1186/1745-6215-11-90 PMC2955653

[11] Haake M, Muller HH, Schade-Brittinger C, Basler HD, Schafer H, et al (2007) German Acupuncture Trials (GERAC) for chronic low back pain: randomized, multicenter, blinded, parallel-group trial with 3 groups. Arch Intern Med 167: 1892–8. 17893311 10.1001/archinte.167.17.1892

[12] Vas J, Mendez C, Perea-Milla E, Vega E, Panadero MD, et al (2004) Acupuncture as a complementary therapy to the pharmacological treatment of osteoarthritis of the knee: randomised controlled trial. Br.Med.J 329(1468-5833): 1216. 15494348 10.1136/bmj.38238.601447.3A PMC529365

[13] Vas J, Perea-Milla E, Méndez C, Sánchez Navarro C, León Rubio JM, et al (2006) Efficacy and safety of acupuncture for chronic uncomplicated neck pain: a randomised controlled study. Pain 126(1–3): 245–55. 16934402 10.1016/j.pain.2006.07.002

[14] Vas J, Ortega C, Olmo V, Perez-Fernandez F, Hernandez L, et al (2008) Single-point acupuncture and physiotherapy for the treatment of painful shoulder: a multicentre randomized controlled trial. Rheumatology (Oxford) 47(6): 887–93. 18403402 10.1093/rheumatology/ken040

[15] Streitberger K, Kleinhenz J (1998) Introducing a placebo needle into acupuncture research. Lancet 352(0140–6736): 364–5. 9717924 10.1016/S0140-6736(97)10471-8

[16] Lund I, Lundeberg T (2006) Are minimal, superficial or sham acupuncture procedures acceptable as inert placebo controls? Acupuncture in Medicine 24(1): 13–5. 16618044 10.1136/aim.24.1.13

[17] Birch S (2006) A review and analysis of placebo treatments, placebo effects, and placebo controls in trials of medical procedures when sham is not inert. J Alternat Complement Med 12: 303–10. 10.1089/acm.2006.12.303 16646730

[18] Hróbjartsson A, Gøtzsche PC (2010) Placebo interventions for all clinical conditions. Cochrane Database Syst Rev. CD003974. 10.1002/14651858.CD003974.pub3 PMC7156905 20091554

[19] Kaptchuk TJ, Stason WB, Davis RB, Legedza ATR, Schnyer RN, et al (2006) Sham device vs. inert pill: randomised controlled trial of two placebo treatments. BMJ 332: 391–7. 16452103 10.1136/bmj.38726.603310.55 PMC1370970

[20] Linde K, Niemann K, Meissner K (2010) Are sham acupuncture interventions more effective than (other) placebos? A re-analysis of data from the Cochrane review on placebo effects. Forsch Komplementrmed 17: 259–64. 10.1159/000320374 20980765

[21] Witt CM, Aickin M, Baca T, Cherkin D, Haan MN, et al (2012) Effectiveness guidance document (EGD) for acupuncture research - a consensus document for conducting trials. BMC Complementary and Alternative Medicine 12(1): 148. 22953730 10.1186/1472-6882-12-148 PMC3495216

[22] Birch S, Felt R (1999) Understanding Acupuncture. Edinburgh: Churchill Livingstone.

[23] Vickers AJ, Rees RW, Zollman CE, McCarney R, Smith CM, et al (2004) Acupuncture for chronic headache in primary care: large, pragmatic, randomised trial. Br.Med.J 328(1468–5833): 744. 15023828 10.1136/bmj.38029.421863.EB PMC381326

[24] Foster NE, Thomas E, Barlas P, Hill JC, Young J, et al (2007) Acupuncture as an adjunct to exercise based physiotherapy for osteoarthritis of the knee: randomised controlled trial. BMJ 335: 436. 17699546 10.1136/bmj.39280.509803.BE PMC1962890

[25] Melchart D, Streng A, Hoppe A, Brinkhaus B, Witt C, et al (2005) Acupuncture in patients with tension-type headache: randomised controlled trial. BMJ 331: 376–82. 16055451 10.1136/bmj.38512.405440.8F PMC1184247

[26] Scharf H, Mansmann U, Streitberger K, Witte S, Kramer J, et al (2006) Acupuncture and Knee Osteoarthritis: a three-armed randomized trial. Ann Intern Med 145: 12–20. 16818924 10.7326/0003-4819-145-1-200607040-00005

[27] Endres HG, Böwing G, Diener H-C, Lange S, Maier C, et al (2007) Acupuncture for tension-type headache: a multicentre, sham-controlled, patient-and observer-blinded, randomised trial. J Headache Pain 8(5): 306–14. 17955168 10.1007/s10194-007-0416-5 PMC3476149

[28] Witt C, Brinkhaus B, Jena S, Linde K, Streng A, et al (2005) Acupuncture in patients with osteoarthritis of the knee: a randomised trial. Lancet 366: 136–43. 16005336 10.1016/S0140-6736(05)66871-7

[29] Brinkhaus B, Witt CM, Jena S, Linde K, Streng A, et al (2006) Acupuncture in patients with chronic low back pain: a randomized controlled trial. Arch Intern Med 166: 450–7. 16505266 10.1001/archinte.166.4.450

[30] Guerra de Hoyos JA, Andres Martin MdC, Bassas y Baena de Leon E, Vigára Lopez M, Molina López T, et al (2004) Randomised trial of long term effect of acupuncture for shoulder pain. Pain 112(3): 289–98. 15561384 10.1016/j.pain.2004.08.030

[31] Kennedy S, Baxter GD, Kerr DP, Bradbury I, Park J, et al (2008) Acupuncture for acute non-specific low back pain: a pilot randomised non-penetrating sham controlled trial. Complement Ther Med 16(3): 139–46. 18534326 10.1016/j.ctim.2007.03.001

[32] White P, Lewith G, Prescott P, Conway J (2004) Acupuncture versus placebo for the treatment of chronic mechanical neck pain: a randomized, controlled trial. Ann.Intern.Med 141(1539-3704): 911–9. 15611488 10.7326/0003-4819-141-12-200412210-00007

[33] Thomas KJ, MacPherson H, Thorpe L, Brazier J, Fitter M, et al (2006) Randomised controlled trial of a short course of traditional acupuncture compared with usual care for persistent non-specific low back pain. BMJ 333: 623–6. 16980316 10.1136/bmj.38878.907361.7C PMC1570824

[34] Salter GC, Roman M, Bland MJ, MacPherson H (2006) Acupuncture for chronic neck pain: a pilot for a randomised controlled trial. BMC Musculoskelet Disord 7: 99. 17156464 10.1186/1471-2474-7-99 PMC1713236

[35] Cherkin DC, Eisenberg D, Sherman KJ, Barlow W, Kaptchuk TJ, et al (2001) Randomized trial comparing traditional Chinese medical acupuncture, therapeutic massage, and self-care education for chronic low back pain. Arch. Intern. Med 161(8): 1081–8. 11322842 10.1001/archinte.161.8.1081

[36] Williamson L, Wyatt MR, Yein K, Melton JT (2007) Severe knee osteoarthritis: a randomized controlled trial of acupuncture, physiotherapy (supervised exercise) and standard management for patients awaiting knee replacement. Rheumatology (Oxford) 46(9): 1445–9. 17604311 10.1093/rheumatology/kem119

[37] Witt CM, Jena S, Brinkhaus B, Liecker B, Wegscheider K, et al (2006) Acupuncture in patients with osteoarthritis of the knee or hip: a randomized, controlled trial with an additional nonrandomized arm. Arthritis Rheum 54: 3485–93. 17075849 10.1002/art.22154

[38] Witt CM, Jena S, Selim D, Brinkhaus B, Reinhold T, et al (2006) Pragmatic randomized trial evaluating the clinical and economic effectiveness of acupuncture for chronic low back pain. Am.J.Epidemiol 164: 487–96. 16798792 10.1093/aje/kwj224

[39] Jena S, Witt CM, Brinkhaus B, Wegscheider K, Willich SN (2008) Acupuncture in patients with headache. Cephalalgia 28(9): 969–79. 18624803 10.1111/j.1468-2982.2008.01640.x

[40] Witt CM, Jena S, Brinkhaus B, Liecker B, Wegscheider K, et al (2006) Acupuncture for patients with chronic neck pain. Pain 125: 98–106. 16781068 10.1016/j.pain.2006.05.013

