# Supplementary material for: Influence of Control Group on Effect Size in Trials of Acupuncture for Chronic Pain: A Secondary Analysis of an Individual Patient Data Meta-Analysis
Source: PLoS One. 2014 Apr 4;9(4):e93739. doi: 10.1371/journal.pone.0093739 (PMC3976298; doi:10.1371/journal.pone.0093739)
Supplement: Table S1 — Sensitivity analyses. (DOCX) [file pone.0093739.s001.docx]

Supporting Information

Table S1: Sensitivity analyses

|  | Excluding Vas trials[12][13][14] and trials with “intermediate likelihood of unblinding”[6][7][8][9] | | |
| --- | --- | --- | --- |
|  | No. of Trials | Change in Effect Size | p value |
| Needle vs. Non-needle sham | 12 vs. 1 | -0.12 (-0.63, 0.38) | 0.6 |
| Non-penetrating needle vs. Non-needle sham | 4 vs. 1 | 0.07 (-0.85, 0.98) | 0.9 |
| Penetrating needle vs. Non-penetrating needle | 8 vs. 4 | -0.20 (-0.50, 0.10) | 0.2 |
| Penetrating needle vs. Non-needle sham | 8 vs. 1 | -0.18 (-0.43, 0.17) | 0.3 |
| Penetrating needle vs. Non-needle sham or Non-penetrating needle | 8 vs. 5 | -0.19 (-0.45, 0.07) | 0.2 |

|  | Including Vas trials[12][13][14] but excluding trials with “intermediate likelihood of unblinding” [6][7][8][9] | | |
| --- | --- | --- | --- |
|  | No. of Trials | Change in Effect Size | p value |
| Needle vs. Non-needle sham | 15 vs.1 | 0.08 (-0.93, 1.10) | 0.9 |
| Non-penetrating needle vs. Non-needle sham | 7 vs. 1 | 0.40 (-0.87, 1.67) | 0.5 |
| Penetrating needle vs. Non-penetrating needle | 8 vs. 7 | -0.57 (-0.98, -0.15) | 0.007 |
| Penetrating needle vs. Non-needle sham | 8 vs. 1 | -0.18 (-0.54, 0.17) | 0.3 |
| Penetrating needle vs. Non-needle sham or Non-penetrating needle | 8 vs. 8 | -0.52 (-0.92, -0.12) | 0.01 |
